# Data collection with a tailored X-ray beam size at 2.69 Å wavelength (4.6 keV): sulfur SAD phasing of Cdc23^Nterm^

**DOI:** 10.1107/S2059798315010268

**Published:** 2016-03-01

**Authors:** Michele Cianci, Matthew R. Groves, David Barford, Thomas R. Schneider

**Affiliations:** a EMBL, Notkestrasse 85, 22607 Hamburg, Germany; b Structural Biology Unit, Department of Pharmacy, University of Groningen, Antonius Deusinglaan 1, 9713 AV Groningen, The Netherlands; c Division of Structural Biology, Institute of Cancer Research, Chester Beatty Laboratories, 237 Fulham Road, London SW3 6JB, England

**Keywords:** SAD phasing, sulfur, soft X-rays, long wavelength, low resolution

## Abstract

The capability to reach wavelengths of up to 3.1 Å at the newly established EMBL P13 beamline at PETRA III, the new third-generation synchrotron at DESY in Hamburg, provides the opportunity to explore very long wavelengths to harness the sulfur anomalous signal for phase determination. Data collection at λ = 2.69 Å (4.6 keV) allowed the crystal structure determination by sulfur SAD phasing of Cdc23^Nterm^, a subunit of the multimeric anaphase-promoting complex (APC/C). At this energy, Cdc23^Nterm^ has an expected Bijvoet ratio 〈|*F*_anom_|〉/〈*F*〉 of 2.2%, with 282 residues, including six cysteines and five methionine residues, and two molecules in the asymmetric unit (65.4 kDa; 12 Cys and ten Met residues). Selectively illuminating two separate portions of the same crystal with an X-ray beam of 50 µm in diameter allowed crystal twinning to be overcome. The crystals diffracted to 3.1 Å resolution, with unit-cell parameters *a* = *b* = 61.2, *c* = 151.5 Å, and belonged to space group *P*4_3_. The refined structure to 3.1 Å resolution has an *R* factor of 18.7% and an *R*_free_ of 25.9%. This paper reports the structure solution, related methods and a discussion of the instrumentation.

## Abstract 2

Data collection with a tailored 50 µm diameter X-ray beam at 4.6 keV (λ = 2.69 Å) on the newly established EMBL beamline P13 at PETRA III allowed the crystal structure determination of the Cdc23^Nterm^ homodimer (65.4 kDa; 12 Cys and ten Met residues) by sulfur SAD phasing at 3.1 Å resolution while overcoming crystal twinning.

## 1. Introduction   

Choosing the wavelength to perform a sulfur SAD (S-SAD) experiment requires thorough consideration. Early landmark S-SAD experiments on crambin (Hendrickson & Teeter, 1981 ▸) and lysozyme (Dauter *et al.*, 1999 ▸) were performed at a wavelength of 1.54 Å using a rotating-anode generator or a synchrotron, respectively, as an X-ray source. During the last decade, the availability of widely tunable beamlines around the world has allowed the use of longer wavelengths or softer X-rays (Djinovic Carugo *et al.*, 2005 ▸; Table 1 ▸). Novel structures such as apo crustacyanin C1 (Gordon *et al.*, 2001 ▸), trypa­redoxin II from *Crithidia fasciculata* (Micossi *et al.*, 2002 ▸) and an IGF2R fragment (Brown *et al.*, 2002 ▸) have been phased by S-SAD at 1.77 Å wavelength at the ESRF, France. At DORIS III, DESY, Germany, data were collected using an X-ray wavelength of 1.74 Å to solve the structure of the nucleocapsid protein of *Porcine reproductive and respiratory syndrome virus* (PRRSv; Doan & Dokland, 2003 ▸). At the SRS, Daresbury, England, an S-SAD data set collected at a wavelength of 2.0 Å, in combination with a xenon-derivative set, was used to provide phase information for apo crustacyanin A1 (Cianci *et al.*, 2001 ▸). At XRD1 Elettra, Italy, the structure of DsvC was solved by collecting data at a wavelength of 1.9 Å (Weiss *et al.*, 2004 ▸). On the X12C beamline at the National Synchrotron Light Source (Brookhaven National Laboratory, Upton, New York, USA), the structure of an FMN reductase from *Pseudomonas aeruginosa* PA01 was determined using an S-SAD data set collected at a wavelength of 1.7 Å (Agarwal *et al.*, 2006 ▸). The structure of VEGF-E was determined by S-SAD using a data set collected at a wavelength of 1.7 Å at the SLS, Villigen, Switzerland (Wagner *et al.*, 2006 ▸). On the PROXIMA 1 beamline at the SOLEIL synchrotron, Saint Aubin, France, *de novo* chlorine/sulfur SAD phasing of a structural protein from ATV was achieved by collecting data at a wavelength of 2 Å (Goulet *et al.*, 2010 ▸).

Long wavelengths of up to 2.65 Å have been tested at XRD1, Elettra, Italy in an extensive series of experiments (Weiss *et al.*, 2001 ▸; Mueller-Dieckmann *et al.*, 2004 ▸, 2005 ▸), with the final conclusion that the optimal data-collection wavelength at which the highest anomalous signal-to-noise ratio could be measured, as assessed by the quotient *R*_anom_/*R*_r.i.m._, was around 2.0–2.1 Å and was independent of any specimen parameter.

More recently, on beamline I03 at Diamond, Didcot, England, successful Cu-SAD experiments on crystals of *Achromobacter cycloclastes* nitrite reductase were performed using the longest X-ray wavelength then available (2.4 Å; Doutch *et al.*, 2012 ▸). At the Photon Factory, KEK, Japan, a comparative study was performed on data collected from death receptor 6 at wavelengths of 2.0 and 2.7 Å to explore the feasibility of S-SAD phasing at longer wavelengths than 2.5 Å. Ru *et al.* (2012 ▸) used a wavelength of 2.7 Å to phase the 160-residue cysteine-rich domain of a tumour necrosis factor using data to 2.95 Å resolution. This showed the potential of very long wavelengths for S-SAD phasing. Liu *et al.* (2001 ▸) investigated the anomalous scattering properties of uranium at its *M*_IV_ (3.326 Å) and *M*_V_ (3.490 Å) edges for multiwavelength anomalous diffraction analysis using crystals of porcine elastase derivatized with uranyl nitrate. Wavelengths of up to 6.0 Å have also been used with the aim of obtaining anomalous diffraction information from the naturally present P and S atoms in biological macromolecules (Stuhrmann *et al.*, 1995 ▸, 1997 ▸; Behrens *et al.*, 1998 ▸). At Diamond, a new all-*in-vacuo* beamline is currently under commissioning, which will be capable of performing crystallographic experiments at wavelengths of up to 4 Å (Allan *et al.*, 2015 ▸; Wagner *et al.*, 2016 ▸).

Besides the accessible energy range at the beamline, other experimental parameters play an important role in choosing the optimal wavelength for an experiment. These parameters, which are related to the nature of the sample, include the Bijvoet ratio 〈|*F*_anom_|〉/〈*F*〉, the allowed X-ray absorbed dose, the diffraction resolution of the crystals and the solvent content (Ramagopal *et al.*, 2003 ▸; Watanabe *et al.*, 2005 ▸; Cianci *et al.*, 2008 ▸). For instance, Agarwal *et al.* (2006 ▸) reported that they decided to collect sulfur SAD data at λ = 1.7 Å since the diffraction quality of their crystal was excellent, *i.e* extending to 1.3 Å resolution, to find a compromise between the resolution and the Bijvoet ratio. Wagner *et al.* (2006 ▸) chose a wavelength of 1.7 Å on the basis of the in-house experience that the expected Bijvoet ratio 〈|*F*_anom_|〉/〈*F*〉 of 1.5%, as estimated from the nature of the sample, would be sufficient for a successful structure solution. In their words, this wavelength would represent a balance between undesired absorption effects and the strength of the anomalous signal. Similar arguments have been reported by Liu *et al.* (2005 ▸). The data resolution and the fractional solvent content are also important factors in phasing using the S-SAD method, where, in general, for crystals with a high solvent fraction and/or higher resolution the density-modified SAD phases are improved and model-building algorithms are more likely to trace the chain residues (Watanabe *et al.*, 2005 ▸).

Hence, consideration of the Bijvoet ratio, the allowed X-ray absorbed dose, the diffraction resolution of the crystals and the solvent content is often critical for successful structure solution using S-SAD (Liu *et al.*, 2005 ▸; Cianci *et al.*, 2008 ▸; Ru *et al.*, 2012 ▸; Weinert *et al.*, 2015 ▸). Liu *et al.* (2005 ▸) developed a ‘parameter-space screening’ method with a high-throughput (HT) structure-determination pipeline. Cianci *et al.* (2008 ▸) proposed an empirical workflow to guide the planning of an S-SAD experiment, which relates the Bijvoet ratio 〈|*F*_anom_|〉/〈*F*〉, the diffraction resolution of the crystals during the experiment, the absorbed dose and the redundancy needed. Similarly, at SLS (Switzerland) a robust data-collection protocol for native SAD phasing has been developed. The protocol foresees the distribution of a tolerable X-ray dose (up to 5 MGy) over high-redundancy data sets (typically 8 × 360°) taken at multiple orientations of a single crystal. All data sets are measured data from a single crystal, using an X-ray wavelength of 2.06 Å (6 keV) as a compromise between sample absorption, maximum attainable resolution and sulfur anomalous signal strength (*f*′′ = 0.96 e; Weinert *et al.*, 2015 ▸). Liu *et al.* (2012 ▸) also estimated the wavelength for the optimum transmitted anomalous signal as a function of X-ray energy and sample size on the basis of X-ray absorption and incoherent scattering. For typical sample sizes of 200, 100 and 50 µm the optimum X-ray energy would be approximately 2.06 Å (6 keV), 2.47 Å (5 keV) and 3.1 Å (4 keV), respectively. Liu *et al.* (2012 ▸) demonstrated the benefits of multi-crystal data collection for an S-SAD experiment by using the strategy to solve several crystal structures at medium to low resolution. Perhaps the most representative result of them all is the 1148-residue TorT/TorS_S_ sensor system, with a Bijvoet ratio 〈|*F*_anom_|〉/〈*F*〉 of 0.83%, which was solved at 2.8 Å resolution.

The EMBL macromolecular crystallography beamline P13 is part of the European Molecular Biology Laboratory integrated infrastructure for life-science applications at PETRA III, the new third-generation synchrotron at DESY in Hamburg. P13 is currently tunable over the energy range from 4 to 17.5 keV to allow crystallographic data acquisition at a broad range of elemental absorption edges for experimental phase determination. The beamline provides a collimated beam [0.2 mrad (horizontal) × 0.15 mrad (vertical)] with a nominal beam size (FWHM) of 30 × 24 µm (horizontal × vertical) and variable focus size (from 30 to 150 µm horizontally and from 24 to 70 µm vertically). The capabilities of P13 to deliver X-rays at long wavelength warranted the consideration of an S-SAD experiment to obtain phase information for the Cdc23^Nterm^ structure.

Cdc23 is one subunit of the multimeric anaphase-promoting complex (APC/C) which controls sister-chromatid segregation and the exit from mitosis by mediating the ubiquitin-dependent degradation of cell-cycle regulatory proteins (Lamb *et al.*, 1994 ▸). The sequence identity of Cdc23^Nterm^ to other proteins is below 30%, making difficult to find models in the PDB for molecular replacement. The Cdc23^Nterm^ subunit has 282 residues, with six cysteines and five methionines (one S atom per 25 residues), and is thus comparable in size and sulfur content to the lysosomal protein from mouse solved using S-SAD by Lakomek *et al.* (2009 ▸). The crystal structure of Cdc23^Nterm^ was solved at higher resolution by Se-SAD in a parallel study by Zhang *et al.* (2013 ▸) and is fully reported there. In this paper, we report the structure solution of Cdc23^Nterm^ achieved at P13 using a S-SAD experiment at λ = 2.69 Å (4.6 keV) and the related methods and we discuss the instrumentation.

## 2. Materials and methods   

### 2.1. Sample preparation, crystallization and crystal mounting   

Cloning, expression and purification of Cdc23^Nterm^ followed the protocol described by Zhang *et al.* (2013 ▸). Crystals were grown by vapour diffusion in a buffer consisting of 200 m*M* sodium formate, 20%(*w*/*v*) polyethylene glycol 3350 and were transferred into a cryoprotection buffer consisting of 200 m*M* sodium formate, 22%(*w*/*v*) polyethylene glycol 3350, 25% glycerol prior to cooling in liquid nitrogen (Zhang *et al.*, 2013 ▸). Crystals of Cdc23^Nterm^ were mounted in a Molecular Dimensions LithoLoop on a SPINE base and flash-cooled in-house for shipping to the synchrotron.

### 2.2. Data collection, processing and structure refinement   

**Figure 1:**
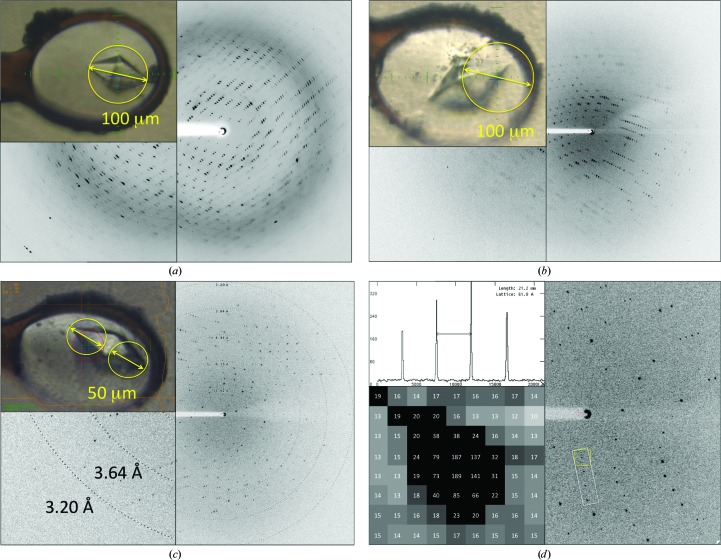
Examples of diffraction images collected at 4.6 keV from Cdc23^Nterm^ crystals of different quality. The images from the Rayonix 225HE detector were displayed with *ADXV* (Arvai, 2015 ▸) using identical contrast levels. The photographs of crystals (*a*, *b*, *c*) were taken with the online camera of the EMBL–Maatel MD2 difffractometer. The yellow circle indicates the beam centre and beam diameter. (*a*) Illuminating the entire volume of a crystal with a collimated 100 µm diameter X-ray beam. (*b*) Illuminating one part with a collimated 100 µm diameter X-ray beam. (*c*) Illuminating one part of the crystal, which gave the data sets, with a collimated 50 µm diameter X-ray beam. (*d*) The low level of X-ray background obtained at P13 when collecting data at λ = 2.69 Å, as shown in a randomly chosen diffraction image. The inserts show pixel values in the region of the diffraction spot (marked in the yellow square) and the relative pixel counts for the spot series (in the white rectangle).

Data for Cdc23^Nterm^ crystals were collected on beamline P13, equipped with a Rayonix 225HE detector, using a collimated X-ray beam of 50–100 µm diameter at 2.69 Å wavelength (4.6 keV) in air. Single octahedrally shaped crystals (Fig. 1 ▸) were mounted on a Maatel MD2 diffractometer and exposed to a nitrogen stream at 100 K. After screening several samples, a crystal was found that diffracted to 3.1 Å resolution with unit-cell parameters *a* = *b* = 61.24, *c* = 151.55 Å and a final space-group assignment of *P*4_3_. The asymmetric unit consists of two molecules, giving a solvent content of 53% and a Matthews coefficient of 2.62 Å^3^ Da^−1^.

The crystal mosaic spread was 0.35° (Table 2 ▸), so using the formula Δφ = 180 × resolution/π × (unit-cell dimension parallel to the beam) (Dauter, 1999 ▸), with a resolution of 3.1 Å and a unit-cell dimension of 61 or 151 Å, the maximum Δφ range was calculated to be between 0.83 and 2°. Considering the morphology of the crystal and its alignment on the loop, we estimated that the fourfold crystallographic axis was nearly aligned along the spindle with a* along the beam (Fig. 1 ▸). A 1° rotation was thus considered to be a good starting value.

All data were integrated using *XDS* (Kabsch, 2010 ▸). For the integration step with *XDS* the keywords ‘FRIEDEL’S_LAW=FALSE’ and ‘STRICT_ABSORPTION_CORRECTION=TRUE’ were used. Space-group determination and consistent assignments of origin were performed using *POINTLESS* (Evans, 2006 ▸). Data were scaled using *SCALA* (Evans, 2006 ▸). For scaling, the ‘ON ROTATION AXIS WITH SECONDARY BEAM ABSORPTION CORRECTION’ protocol was used. Data-collection and refinement statistics are reported in Table 2 ▸.

### 2.3. Phasing and structure refinement   

The merged data from the data sets collected from the two tips of one crystal were input to *SHELXC* (Sheldrick, 2008 ▸) and *SHELXD* (Schneider & Sheldrick, 2002 ▸) for sulfur-substructure determination using *HKL*2*MAP* (Pape & Schneider, 2004 ▸). The solution with the highest correlation coefficient (CC) between the observed and calculated Bijvoet differences was used with *Phaser* for SAD applications (Read & McCoy, 2011 ▸) for site refinement and phasing. Automated model tracing was achieved with the *PHENIX AutoBuild* module (Adams *et al.*, 2010 ▸). The starting model was manually rebuilt with *Coot* (Emsley *et al.*, 2010 ▸) and refined using the *PHENIX* package (Adams *et al.*, 2010 ▸) and the *PDB_REDO* webserver (Joosten *et al.*, 2014 ▸). Randomly selected reflections (5% of the total) were used as an *R*_free_ set for cross-validation. Refinement converged to a final *R* factor and *R*_free_ of 18.7 and 25.9%, respectively. The stereochemistry of the final model was checked using *Coot* (Emsley *et al.*, 2010 ▸) and *PROCHECK* (Winn *et al.*, 2011 ▸). Graphics were generated with *CCP*4*mg* (McNicholas *et al.*, 2011 ▸). The *RADDOSE* (Paithankar *et al.*, 2009 ▸) parameters used for dose estimation were a photon flux of 1.51 × 10^8^ photons s^−1^ (measured flux at the beamline; see §[Sec sec3.1]3.1), an exposure time of 20 s, 360 images per degree of rotation, beam size = crystal size = 50 µm and 564 residues in total with 22 S atoms.

## 3. Results and discussion   

### 3.1. Instrumentation   

The PETRA III storage ring at DESY, Hamburg, Germany provides a low-divergence X-ray beam. The P13 beamline design minimizes the X-ray scattering background by realising an all-in-vacuum design up to 50 mm from the sample position. The Rayonix 225HE detector was fitted with a customized phosphor thickness of 25 µm to increase the signal-to-noise ratio by limiting the point-spread function and a backlit CCD chip to increase the efficiency at low energies. Despite working in an ambient atmosphere, no evidence of strong air scatter was seen in the diffraction images (Fig. 1 ▸). The Maatel–EMBL MD2 diffractometer (Perrakis *et al.*, 1999 ▸), with the pre-fitted set of apertures, allowed cleaning and shaping of the beam to the size of the crystal (Fig. 1 ▸). The endogenous background is further minimized (Fig. 1 ▸ *d*) with a scatter guard and a metal capillary mounted on the MD2 diffractometer. The high flux at low energies measured at P13 (>2.3 × 10^11^ photons s^−1^ at 2.69 Å wavelength; 4.6 keV) with an unfocused beam (1 × 2 mm, vertical × horizontal) allowed the beam to be collimated to ∼50 µm in diameter (photon flux of 1.51 × 10^8^ photons s^−1^), while still collecting two complete data sets (one at each end of the crystal) in around 4 h.

It should be noted that P13 now operates at long wavelengths with a Dectris Pilatus 6M detector with 450 µm sensor thickness, custom calibration tables for low energies and with a constant helium flow for the detector case and the helium cone. The beamline thus maintains its efficiency in working at long wavelengths, also benefiting from the small point-spread function, low readout noise and high frame rate of the Pilatus 6M detector. Data-collection times on P13 are in fact reduced to a few minutes when the beamline is operated with a fully focused beam [>2.3 × 10^11^ photons s^−1^ at 2.69 Å wavelength (4.6 keV) in a 30 × 24 µm (horizontal × vertical) spot].

### 3.2. Data collection from Cdc23^Nterm^ crystals   

**Figure 2:**
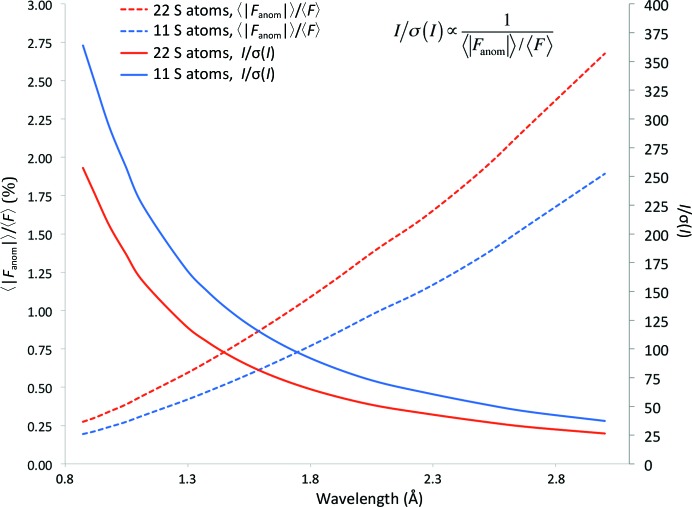
Plot of the expected Bijvoet ratio 〈|*F*_anom_|〉/〈*F*〉 (dashed line) and *I*/σ(*I*) (full line) *versus* wavelength calculated for two monomers of Cdc23^Nterm^ in the asymmetric unit (582 residues) with 22 S atoms (red line) or 11 S atoms (blue line). The values of *f*′′ for sulfur (in eV) used for the calculation were taken from the web server of the Biomolecular Structure Center at the University of Washington School of Medicine (http://www.bmsc.washington.edu/scatter/AS_periodic.html).

The wavelength was chosen to maximize the Bijvoet ratio, on the basis of the available energy range at the beamline and the expected diffraction resolution of the sample, following the decision-making model for an S-SAD experiment proposed by Cianci *et al.* (2008 ▸). For the expected resolution limit of the crystals of around 3.0 Å, the corresponding maximum diffraction angles could be collected on the Rayonix 225HE detector (active area 225 × 225 mm) using X-rays at 2.69 Å wavelength (4.6 keV) combined with the minimum crystal-to-detector distance of 90 mm. With this wavelength the expected value of 〈|*F*_anom_|〉/〈*F*〉 for Cdc23^Nterm^ with six Cys residues and five Met residues in 282 residues would be enhanced from 1.0% at 7 keV (1.7 Å wavelength) to 2.2% (Fig. 2 ▸).

According to Liu *et al.* (2012 ▸), the optimum wavelength for the transmitted anomalous signal, as a function of X-ray energy, for crystals of 100 µm thickness would be between 5 and 4 keV. For crystals of 50 µm thickness the transmitted anomalous signal would be maximal at 4 keV, *i.e.* at a wavelength of 3.1 Å. Thus, given the size of the Cdc23^Nterm^ crystals, which were up to 100 µm in the longest axis, the use of this wavelength was deemed to be appropriate (Liu *et al.*, 2012 ▸, 2013 ▸).

Screening of several crystals was required before one was found that was suitable for data collection. Despite all of them being of similar size, mounted with the same type of loop and soaked in cryoprotectant solution, the diffraction varied substantially between samples (Fig. 1 ▸). Some samples showed higher background noise, higher crystal mosaicity and lower resolution than others. The importance of paying attention to crystal mounting for a low-energy SAD experiment have been clearly described by Kitago *et al.* (2005 ▸), where minimization, if not complete removal, of the solution surrounding the crystal greatly enhanced the *I*/σ(*I*) and 〈|Δ*F*|/σ(Δ*F*)〉 of the collected data sets, thus benefiting the complete process from substructure determination to phasing.

**Figure 3:**
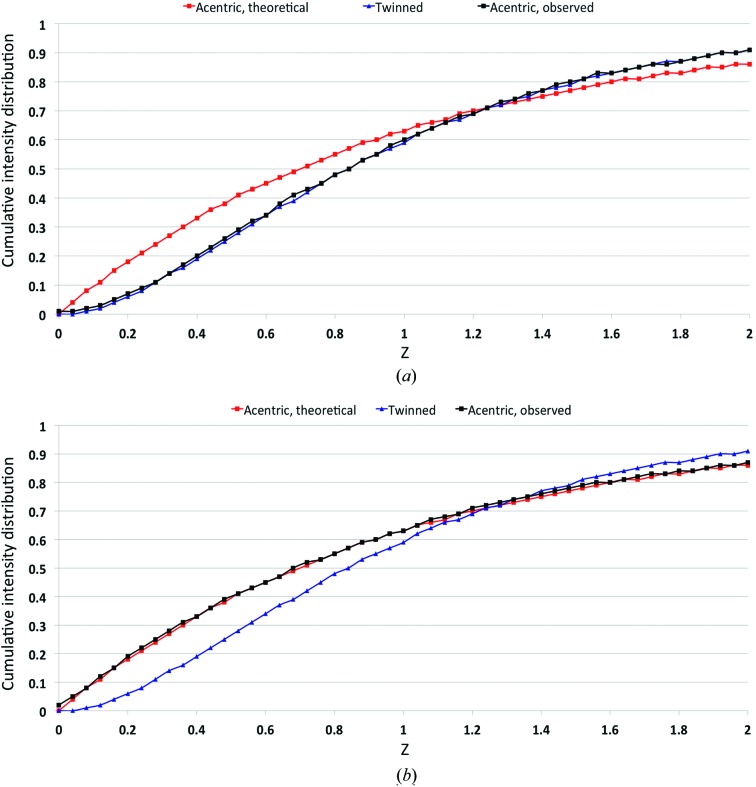
Plot of the cumulative intensity distribution *Z* for acentric reflections from scaling the data using *SCALA* (Evans, 2006 ▸) (*a*) for a data set collecting by illuminating the whole crystal volume and producing twinned data and (*b*) for the final untwinned data obtained by merging the two data sets collected separately by illuminating the two tips of the octahedral crystal.

Initial data were collected from a single prefrozen crystal of octahedral shape entirely exposed to a collimated X-ray beam of 100 µm in diameter (Fig. 1 ▸ *a*). *POINTLESS* (Evans, 2006 ▸) suggested a space-group assignment of *P*4_1_22. The plot of the cumulative intensity distribution *Z*, as determined by *TRUNCATE* (Winn *et al.*, 2011 ▸), for the scaled data indicated significant deviation from the expected behaviour for untwinned data (Fig. 3 ▸ *a*). As no twin laws are possible for this crystal symmetry, the data were reprocessed with *P*4_*x*_ symmetry. *TRUNCATE* (Winn *et al.*, 2011 ▸) and *PHENIX* (Adams *et al.*, 2010 ▸) confirmed the presence of merohedral twinning with twin law (*h*, −*k*, −*l*) with an estimated twin fraction of 0.37. Similar twinning problems were experienced and described by Zhang *et al.* (2013 ▸) in the structure solution of the Cdc23^Nterm^ selenomethionine-derivatized crystals.

**Figure 4:**
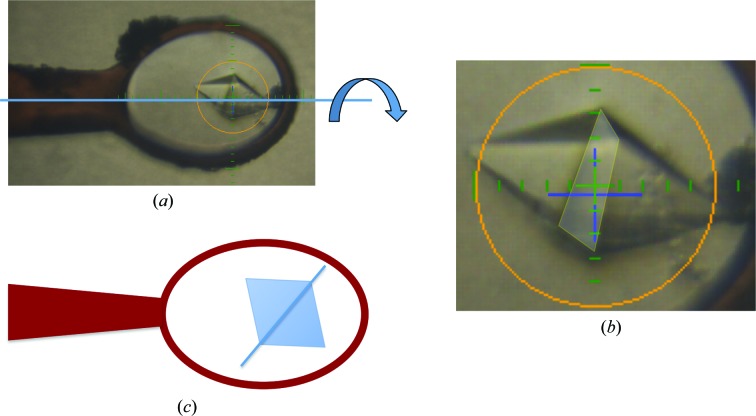
(*a*) Observation of the crystal morphology by spinning the crystal around the φ axis using the online camera of the MD2 diffractometer (Perrakis *et al.*, 1999 ▸). (*b*) The interface plane coinciding with the base plane of the two square pyramids. (*c*) A schematic representation of the two square pyramids, forming an octahedron. The coloured circle has a diameter of 100 µm.

However, in-depth observation of the crystal morphology, using the online camera of the MD2 diffractometer (Perrakis *et al.*, 1999 ▸), while rotating the crystal around the φ axis highlighted the presence of an interface plane coinciding with the base plane of the two square pyramids (Fig. 4 ▸). This observation suggested that the two square pyramids forming the octahedron could be two separate crystalline domains. Thus, for the subsequent data collections each half of the crystal was illuminated separately: firstly with a beam size of 100 µm in diameter (Fig. 1 ▸ *b*) and then with a beam size reduced to 50 µm in diameter to reduce the background owing to the cryo-buffer or the loop structure (Fig. 1 ▸ *c*). After integration, *POINTLESS* (Evans, 2006 ▸) indicated a space-group assignment of *P*4_*x*_. The cumulative intensity distribution plot from scaling the data in *TRUNCATE* (Winn *et al.*, 2011 ▸) did not show any further signs of crystal twinning (Fig. 3 ▸ *b*). Clearly, the two halves of the crystal were actually two separate crystalline domains and were successfully treated as such. The capability to adapt the beam size to selectively illuminate different parts of the sample allowed the crystal imperfections to be overcome. The usefulness of scaling low-energy data with *SCALA* (Evans, 2006 ▸) using the ‘ON ROTATION AXIS WITH SECONDARY BEAM ABSORPTION CORRECTION’ protocol has been discussed previously by Mueller-Dieckmann *et al.* (2004 ▸) and Cianci *et al.* (2008 ▸).

### 3.3. Anomalous signal and structure solution of Cdc23^Nterm^   

**Figure 5:**
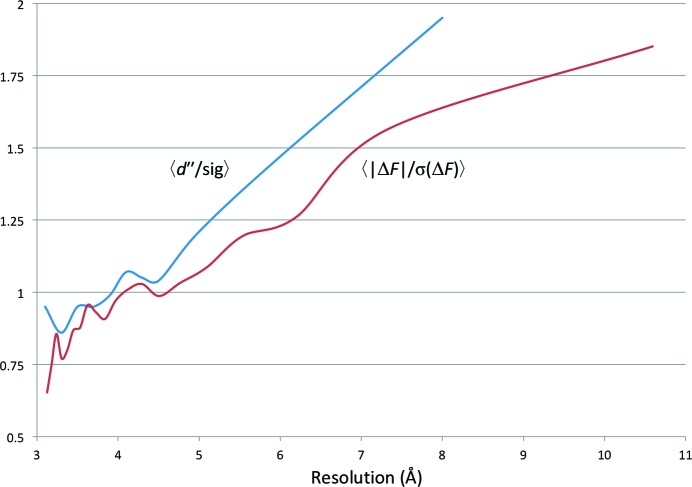
Plot of *d*′′/sig(*d*′′) (blue line) from *SHELXC* (Sheldrick, 2008 ▸) and 〈|Δ*F*|/σ(Δ*F*)〉 calculated with *SFTOOLS* (Winn *et al.*, 2011 ▸; red line) *versus* resolution for the Cdc23^Nterm^ data.

**Figure 6:**
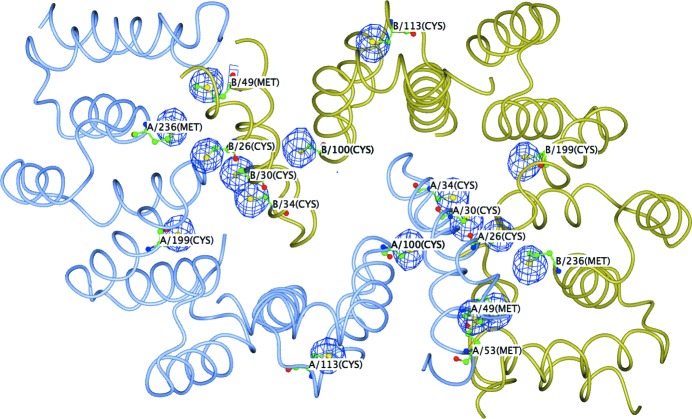
Anomalous difference Fourier map calculated using phases from the *Phaser* solution contoured at the 6σ level superimposed onto the final model.

**Figure 7:**
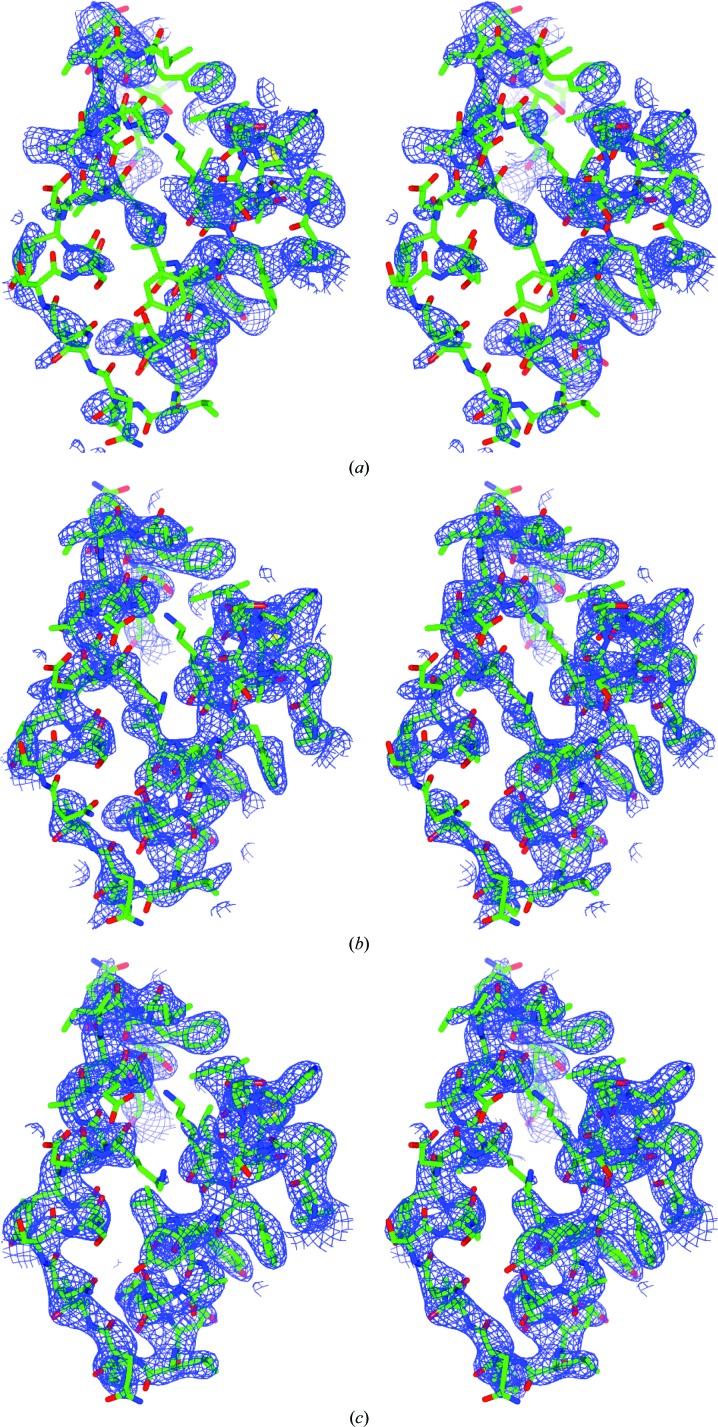
Stereoviews of 2*F*_o_ − *F*_c_ difference Fourier maps with 1.5σ cutoff superimposed on the refined model (chain *A*, residues 230–270). Top, phases from *Phaser*; middle, after model building with *PHENIX*; bottom, after refinement with *PHENIX*.

Analysis of the data with *SHELXC* (Sheldrick, 2008 ▸) indicated the presence of anomalous signal with a 〈|Δ*F*|/σ(Δ*F*)〉 ratio of greater than 1.0 up to 4.0 Å resolution (Fig. 5 ▸). With two monomers in the asymmetric unit, as suggested by the Matthews coefficient, a total of 22 sulfur sites (12 cysteine residues and ten methionine residues) were expected. In fact, a successful *SHELXD* (Schneider & Sheldrick, 2002 ▸) run was obtained (100 attempts with two solutions) with a resolution cutoff at 4.0 Å and searching for 20 S atoms. *SHELXD* identified 17 correct sites (out of the 18 final refined sites, with the missing four located in disordered regions of the structure) and *SITCOM* (Dall’Antonia & Schneider, 2006 ▸) confirmed the presence of an NCS twofold axis. *Phaser* (McCoy *et al.*, 2007 ▸; Read & McCoy, 2011 ▸) with the keyword ‘LLG­COMPLETE COMPLETE ON’ was used to generate the log-likelihood maps to refine the *SHELXD* sites and to produce the initial set of phases for the two hands. The highest peaks in the anomalous difference Fourier map (Fig. 6 ▸) correspond to two disulfide bridges (one per monomer) and 14 single S atoms. Both initial set of phases at 3.1 Å resolution were subsequently used for automated density modification and auto-tracing of the peptide chain with *phenix.autobuild* in *PHENIX* (Adams *et al.*, 2010 ▸), thus identifying the correct hand upon a positive result. The final autobuilt model comprised 384 residues with 28 fragments. The partial *R* factor and *R*_free_ were 27.7 and 35.8%, respectively. In *PHENIX*, *phenix.autobuild* was run with the options ‘find_ncs=Auto’ and ‘optimize_ncs=use_ncs_in_build=refine_with_ncs=True’. Changing to ‘find_ncs=False’ sensibly lowered the quality of the results, with a final rebuilt model of 180 residues only. The r.m.s.d.s between the completely refined substructure and the *SHELXD* and the *Phaser* sites, calculated with *SITCOM* (Dall’Antonia & Schneider, 2006 ▸), are 0.64 and 0.37 Å, respectively. The correlation coefficients between the map calculated with the phases from the final, manually rebuilt model against the maps calculated with the phases after *Phaser* and after *phenix.autobuild* were 42 and 74%, respectively (Fig. 7 ▸). The C^α^ r.m.s. deviations, calculated with *LSQKAB* (Murray *et al.*, 2004 ▸) for 219 residues, between this structure and that reported by Zhang *et al.* (2013 ▸) are 0.80 and 0.92 Å for chain *A* and chain *B*, respectively. The quality of the calculated phases allowed the autotracing of 384 of the final 472 rebuilt residues (81%) at 3.1 Å resolution.

### 3.4. Radiation damage and multiplicity   

**Figure 8:**
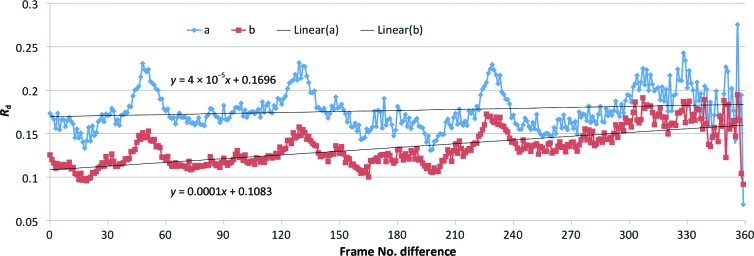
Plot of the ‘decay *R* factor’ *versus* frame-number difference (Diederichs, 2006 ▸) for the two Cdc23^Nterm^ data sets (denoted ‘a’ and ‘b’). Interpolation functions for each data set are indicated on the plot.

The overall X-ray dose for a 360° data collection was estimated with *RADDOSE* (Paithankar *et al.*, 2009 ▸) to be 1.8 MGy, equivalent to ∼1/10 of the Henderson limit (Henderson, 1990 ▸; Owen *et al.*, 2006 ▸). Similar doses have previously been reported to cause radiation damage to disulfide bridges (Wang *et al.*, 2006 ▸; Shimizu *et al.*, 2007 ▸; Cianci *et al.*, 2008 ▸). For the crystal structure of Cdc23^Nterm^ discussed here, no evidence of radiation damage was present in the electron-density maps. In addition to the inspection of electron-density maps, we looked for indications of radiation damage by plotting the ‘decay *R* factor’ *versus* the frame-number difference (Diederichs, 2006 ▸). While a positive slope of the trace would be expected for significant radiation damage, for the two Cdc23^Nterm^ data sets the slope values were close to zero (Fig. 8 ▸). Interestingly, the plot of the ‘decay *R* factor’ for the two Cdc23^Nterm^ data sets shows a regular repetition every 90°. Rather than being a consequence of radiation damage, this feature could be the fingerprint of the absorption through the crystal exposed to the X-ray beam, given its alignment along the spindle axis (Fig. 1 ▸). Similar signatures have been observed previously for lysozyme and apo crustacyanin A1 and were attributed to either absorption or radiation damage (Cianci *et al.*, 2004 ▸). Their presence has been shown to dramatically affect the substructure-location procedure and required special treatment during scaling (Cianci *et al.*, 2004 ▸). An optimal scaling approach, possibly with a crystal-tailored absorption correction, should be able to compensate for such features with data collected at long wavelengths.

With a low X-ray energy of 4.6 keV, the expected Bijvoet ratio 〈|*F*_anom_|〉/〈*F*〉 for Cdc23^Nterm^ with six Cys residues and five Met residues in 282 residues was enhanced from 1.0% at 7 keV (at 1.7 Å wavelength) to 2.2% (Fig. 2 ▸). According to the formula *I*/σ(*I*) = (2^1/2^/2) × (*a*/*q*), where *a* = Δ*F*/σ(Δ*F*) is the signal to noise in the Bijvoet difference and *q* = 〈|*F*_anom_|〉/〈*F*〉 or the Bijvoet difference ratio (Liu *et al.*, 2013 ▸), a value of *I*/σ(*I*) ≃ 32 is needed to achieve a signal to noise of 1 with an expected Bijvoet difference of 2.2%. In fact, the anomalous signal of Cdc23^Nterm^ was measured with two data collections from two regions of one crystal with an overall multiplicity (Friedel pairs were kept separate for merging statistics) of 27 and a final overall 〈*I*/σ(*I*)〉 of 30.1. Following these arguments, it can then be estimated that an S-SAD data collection on Cdc23^Nterm^ at 7 keV would have required an overall value of *I*/σ(*I*) ≃ 70. Overall, the increase in the Bijvoet ratio reduces the value of *I*/σ(*I*) required to achieve successful phasing. Together with the fact that two data sets can be collected from a single crystal within the radiation-damage limits to achieve an *I*/σ(*I*) of 30, it is probable that more than two crystals of this size would have been required for an S-SAD data collection on Cdc23^Nterm^ at 7 keV if they were found to be isomorphous. The reasoning used here is based on some sort of ‘ideal’ behaviour in which the sample, for example, has fully occupied heavy-atom sites or isotropic diffraction. In other words, it would be possible to prepare for a perfectly respectable Bijvoet ratio, but the anomalous scatterers could happen to be in disordered regions of the macromolecule, thus reducing *de facto* the contribution to the anomalous signal 〈|*F*_anom_|〉/〈*F*〉. In this case a higher *I*/σ(*I*) would be required and a higher multiplicity would be needed since *I*/σ(*I*) ∝ 1/(〈|*F*_anom_|〉/〈*F*〉). This scenario is illustrated in Fig. 2 ▸ for the case of Cdc23^Nterm^, where the Bijvoet difference is estimated firstly assuming that all 22 S atoms are present and secondly with only 11 S atoms present owing to the location of the missing S atoms in disordered regions. It can be seen that halving the number of sulfur sites reduces the Bijvoet difference (〈|*F*_anom_|〉/〈*F*〉) requested for a successful experiment by ∼30% and increases the *I*/σ(*I*) by an equal amount. However, since *I*/σ(*I*) ∝ 1/(〈|*F*_anom_|〉/〈*F*〉), the change in the absolute value of the required *I*/σ(*I*) is much smaller when working at long wavelengths compared with shorter wavelengths. Thus, when working with long wavelengths for a S-SAD experiment, the increase in multiplicity necessary to compensate for an underestimated *I*/σ(*I*) is only minor.

Similarly to S atoms, the long-wavelength radiation will also enhance the anomalous signal from P atoms in DNA-binding complexes, or from K or Ca atoms (Table 3 ▸), favouring phasing. For instance, the expected Bijvoet ratio for the T_2_R–TTL complex (Weinert *et al.*, 2015 ▸), which contains 2317 residues with 118 S atoms, 13 P atoms, three Ca atoms and two Cl atoms, is 1.7% at 2.06 Å wavelength (6 keV) and 3.1% at 3.1 Å wavelength (4 keV) as calculated using the *ASSC* (*Anomalous Scattering Signal Calculator*) web server (Olczak *et al.*, 2003 ▸, 2007 ▸; Olczak & Sieron, 2015 ▸).

## 4. Conclusions   

The provision of a 2.69 Å wavelength (4.6 keV) beam with variable beam size was instrumental for the structure solution of Cdc23^Nterm^ at 3.1 Å resolution *via* the anomalous signal from S atoms intrinsic to the protein. A variable micrometre-size beam allowed the collection of sulfur data from separate regions of a crystal, which would otherwise be discarded, thus overcoming crystal imperfections owing to the presence of two differently oriented twin domains. The long wavelength enhanced the Bijvoet ratio to 2.21% (1.0% at 7 keV), thus increasing the anomalous signal to a level where it was possible to determine the sulfur substructure accurately even with moderate data multiplicity. The quality of the calculated phases allowed complete model autotracing at a resolution as low as 3.1 Å.

The capability of P13 to deliver X-rays with wavelengths between 2.06 and 3.1 Å (6–4 keV energy range), and the availability of software packages that are capable of handling data processing and low-resolution phasing, open up new opportunities for sulfur anomalous signal phasing from bio­logical macromolecules, thus contributing to lowering the current limits of a SAD experiment both in terms of sulfur content and of diffraction quality of available crystals, as advocated by Doutch *et al.* (2012 ▸) and Liu *et al.* (2012 ▸, 2013 ▸). Increasing the Bijvoet ratio means reducing the value of *I*/σ(*I*) required to achieve successful phasing, with the advantage of reducing the demand in multiplicity and in crystal supply. S-SAD phasing can be considered as an option for low-resolution phasing.

## Supplementary Material

PDB reference: sulfur SAD phasing of Cdc23 APC subunit, 5ftp

## Figures and Tables

**Table 1:** Trend in the wavelengths used for some successful S-SAD experiments

Protein	Molecular weight (kDa)	Wavelength (Å)	Bijvoet ratio at the wavelength reported (%)	Resolution (Å)	Reference
Crambin	4.74	1.54	1.5	1.5	Hendrickson & Teeter (1981 ▸)
Lysozyme	14	1.54	1.15	1.53	Dauter *et al.* (1999 ▸)
Apo crustacyanin C1	40	1.77	0.96	2.5	Gordon *et al.* (2001 ▸)
Tryparedoxin II	16.9	1.77	1.12	2.35	Micossi *et al.* (2002 ▸)
IGF2R fragment	15.6	1.77	1.47	1.95	Brown *et al.* (2002 ▸)
DsvC	12	1.9	1.4	1.85	Weiss *et al.* (2004 ▸)
PRRSv	8.1	1.74	1.07	2.6	Doan & Dokland (2003 ▸)
FMN reductase	20	1.7	0.76	1.76	Agarwal *et al.* (2006 ▸)
VEGF-E	14.2	1.7	1.5	3.0	Wagner *et al.* (2006 ▸)
Lysosomal protein	66.3	1.9	1.2	2.4	Lakomek *et al.* (2009 ▸)
ATV	14.6	2	1.41	2.7	Goulet *et al.* (2010 ▸)
TNFR-CRD	17.8	2.7	3.93	2.95	Ru *et al.* (2012 ▸)
TorT/TorS_S_	127	1.7	0.83	2.8	Liu *et al.* (2012 ▸)
T_2_R–TTL	266	2.0	1.66	2.3	Weinert *et al.* (2015 ▸)
Cdc23^Nterm^	65.4	2.69	2.21	3.1	This work

**Table 2:** Data-collection, processing and refinement statistics for Cdc23^Nterm^ Values in parentheses are for the highest resolution bin.

Data collection	
Wavelength (Å)	2.69
Photon flux (photons s^−1^)	1.51 × 10^8^
Beam size (diameter) (µm)	50
Detector	Rayonix 225HE
No. of crystals	1 [two halves]
Oscillation angle (°)	1.0
Crystal mosaicity (°)	0.35
Exposure time (s)	20
Crystal-to-detector distance (mm)	90
No. of images	360 + 360
Space group	*P*4_3_
Unit-cell parameters (Å)	*a* = *b* = 61.2, *c* = 151.5
Resolution range (Å)	61.25–3.10 (3.21–3.10)
Total No. of reflections	282255 (25782)
Unique reflections	10165 (1448)
Multiplicity	27.8 (17.8)
Completeness (%)	99.7 (98.0)
*R*_merge_ † (%)	11.4 (72.2)
Mean *I*/σ(*I*)	30.1 (4.8)
Matthews coefficient (Å^3^ Da^−1^)	2.62
Solvent content (%)	53.0
Structure refinement	
*R* factor	0.187
*R*_free_	0.259
No. of atoms	
Total	3865
Macromolecules	3858
Waters	7
No. of protein residues	472
R.m.s.d., bonds‡ (Å)	0.01
R.m.s.d., angles‡ (°)	1.39
Ramachandran plot§	
Most favoured (%)	91.3
Allowed (%)	7.5
Generously allowed (%)	0.7
Outliers (%)	0.5
Average *B* factor‡ (Å^2^)	
Macromolecules	60.1
Solvent	32.5

† *R*_merge_ = 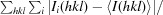 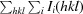, where *I_i_*(*hkl*) is the intensity of the *i*th observation of reflection *hkl* and 〈*I*(*hkl*)〉 is the mean intensity of all *i* symmetry-related reflections.

‡ Values from *PHENIX* (Adams *et al.*, 2010 ▸).

§ Values from *PROCHECK* (Laskowski *et al.*, 1993 ▸).

**Table 3:** Anomalous scattering coefficient *f*′′ (e^−^) for common protein atoms of interest for phasing in the 4–12 keV range The values are taken from http://skuld.bmsc.washington.edu/scatter/AS_periodic.html.

Atom	0.98 Å (12.6 keV)	1.54 Å (8 keV)	2.06 Å (6 keV)	2.47 Å (5 keV)	2.69 Å (4.6 keV)	3.1 Å (4 keV)
Na	0.05	0.12	0.22	0.32	0.37	0.48
P	0.18	0.44	0.75	1.04	1.20	1.52
S	0.24	0.56	0.95	1.31	1.52	1.92
K	0.47	1.07	1.77	2.40	2.75	3.43
Ca	0.57	1.29	2.12	2.83	3.23	0.43
Cl	0.30	0.71	1.19	1.63	1.87	2.36
